# An Ovariotomy

**Published:** 1884-06

**Authors:** Seth N. Jordan

**Affiliations:** Columbus, Ga.


### AN OVARIOTOMY.

BY SETH N. JORDAN, M. D., COLUMBUS, GA.

An ovarian tumor weighing fifty-seven pounds removed, and followed by after-treatment somewhat at variance with the accurate rules prescribed in the text books, may be worth a description, and surely should have a statistical value.

Mrs. E. Davis, of Nance Station, Muscogee county, was admitted into the city hospital September 9th, 1883. *Status presens:* Patient, widow, 31 years of age, mother of three children; measures 41 inches around the abdomen, musculature moderately well developed.

Patient first noticed that she was growing larger about one year ago. Is now suffering to that extent from dyspnœa and palpitation, when in a recumbent posture, that she does not dare lie down. She readily consented to any operation in order to obtain sleep, which had almost deserted her. As the skin over the tumor was quite tense and the palpitation distressing, it was determined to aspirate the tumor at once, trusting thus to improve the general condition in order to perform ovariotomy on the following day. On the 10th, by means of an aspirator, thirty-nine pints of a moderately thick, syrupy fluid was removed from the tumor. The unpleasant symptoms entirely disappeared. Microscopic examination, as well as other diagnostic measures, seemed to leave little doubt as to the nature of the tumor, namely: unilocular ovarian cyst. The strictest antiseptic precautions were adhered to in every respect. Every article of any kind whatsoever was removed from the operating room. It was then thoroughly whitewashed and sprayed over with carbolic acid solution.

The only furniture in the room at the time of the operation was a bed and an operating table. The bed was covered with new oilcloth and new bedding. The instruments had been in carbolic acid solution for several hours.

The anesthetic was administered by the hospital matron. The mixture known as Billroth's.

| | |
|---|---|
| Chloroform, parts by weight . . . . . . . . | 100 |
| Ether, " " " . . . . . . . . . | 30 |
| Alcohol, " " " . . . . . . . . . | 30 |

was used, and is the only anesthetic that I have administered in six years.

Dr. T. P. Chafin was the only assistant, to whom I feel much indebted for his valuable aid. An incision five inches long was made in the linea alba and careful steps were taken until a thick membrane came in view. This proved to be the peritoneum bound solidly to the sack. An incision was made in this which revealed a quantity of fluid. A trocar was thrust within and six pints of syrupy fluid emptied. It was with great difficulty that a point could be found through which an entrance might be gained in order to break down the absolutely uninterrupted adhesions. By working slowly and using much strength, the pedicle was finally reached. Wherever hemorrhage occurred fine silk sutures were employed. A piece of the sack about three by four inches was accidentally torn off and allowed to remain. There were also slight adhesions with the uterus. The pedicle was found to be four inches wide and quite short. On this account it was tied in two sections with stout catgut and burnt into by means of a galvan-caustic loop. It may have been fortunate that the cautery was at hand, as the width and shortness of the pedicle gave reasonable apprehension of after hemorrhage had it been severed by the knife. I cannot conceive of more solid and continuous adhesions on every side than in this case, nor does it now seem that, at any time should an ovariotomy be left uncompleted owing to vastness of adhesions. In spite of torsion several oozing points were left. The toilet of the periotoneum was made with new sponges that had been in a carbolic acid solution for several hours. The abdominal wound was closed up with nine deep silk sutures; sublimate gauze was the dressing used. The operation lasted one hour and forty minutes. The tumor went out from the left ovary and weighed fifty-seven pounds.

Thirty-nine pints removed day previous to operation; six pints during operation; sack twelve pounds. There were numerous small cysts connected with the ovary.

The patient awoke soon after the operation complaining of cold in the entire lower extremities. By means of hot bottles and the hypodermic injection of 1-4 gr. morph. with one hundredth gr. atropia this passed off and she rested well. For three days the temperature never exceeded 101° nor the pulse 105°. During this time small quantities of dry champagne were given. She was constantly nauseated but vomited only once on the second and third days. On the third day small quantities of iced milk were given and retained. On the afternoon of the fourth day the temperature mounted up to 103½°. That night, after the administration of castor oil in an enema, several large, offensive stools passed. The next morning the temperature was down to 101°, afternoon 103°. About thus it continued for seventeen days. The temperature once reached 105°, several times 104°. During the entire period of fever after the third day repeated actions on the bowels followed every night spontaneously. After the bowels had moved a suppository of opium 1 gr., belladonna extract, 1-6 gr., was introduced to produce sleep. At no other time was an opiate or other medicine administered. Tympanitis was marked from the second day. The urine was drawn off every six hours until the ninth day when it passed off itself. Pain was present all the time in the right side immediately over the region the piece of the sack was torn from. It required some fortitude on my part to give no heed to the entreaties for opium, but as the pulse kept its tone without it, it was thought advisable to withhold everything that would impair natural drainage through the intestinal canal. When the temperature reached 105° I determined to introduce a glass drainage tube through the vagina; an operation which I had seen Gusserow, in Strasbourg, perform with advantage. That night, after several stools had passed, the temperature registered 101½. I desisted from the introduction of the tube. The impulse to employ drainage was constantly with me, but I withstood it, always encouraged by the morning's improvement, the tone of the pulse and the patient's ability to retain milk and stimulants.

I regarded the condition as septicæmia, brought about by the fluid that had oozed into the peritoneal cavity. Nature had set up a drainage of this offending substance, and, inspired by the suggestion of Tait, "Diseases of the Ovaries," p. 315, I put no hindrance in the way. I have been but feebly impressed by the action of remedies given internally in septicæmia. I have faith alone in alcohol in this condition, and that does not, to my mind, act specifically but simply retards elimination. Incidentally added: This manner of treatment, withholding febrifuges and opiates, has carried two cases of puerperal septicæmia to a safe terminus for me since my experience above cited. The dressing was not removed until the eighth day, when the stitches were removed. On the fifth day the wound was examined and the gauze readjusted. At the end of the second week a circumscribed induration appeared in the incision, which formed an abscess and discharged slowly. There was a temporary elevation of temperature for a few days. There was apprehension at first that the abscess might go out from the stump. This, however, was not so.

The individualities in this case are: the size of the tumor, being much above the average; the unbroken line of adhesions, and adhesions with the uterus.

The divergence in treatment consists in: Aspiration of the contents of the cyst preceded the ovariotomy one day; the pedicle was tied with catgut in two sections, then severed by the galvano cautery. The after-treatment was almost expectant. Febrifuges—save alcohol—were not administered. Drainage took place entirely through the bowels. And this case fully illustrates nature's powers when left unfettered. It is exceedingly difficult to check all bleeding points in a case with such extensive adhesions, and consequently serum must accumulate in the peritoneum. If nature does not produce the first step to removing such fluid, then it behooves us to induce purgation with this aim. Too often the opposite direction is taken, opium being given in an old-fashioned routine manner to allay a beginning peritonitis.

My patient at this writing weighs 150 pounds, and is strong and robust.

